# Addition of Intravenous Immunoglobulin in Refractory Cutaneous Polyarteritis Nodosa: A Case Report

**DOI:** 10.1155/crrh/4646918

**Published:** 2026-10-06

**Authors:** Fabio Kohlmann, Daniela Odehnal, Laura Buchtala, Anna Kernder, Xenofon Baraliakos

**Affiliations:** ^1^ School of Medicine, Faculty of Health Witten/Herdecke University Witten Germany uni-wh.de; ^2^ Department of Internal Medicine and Rheumatology Rheumazentrum Ruhrgebiet Marien Hospital Herne University Hospital of Ruhr University Bochum Herne Germany; ^3^ Department of Pediatrics and Adolescent Medicine Prof. Hess Children’s Center Klinikum Bremen-Mitte Bremen Germany

**Keywords:** case report, cutaneous polyarteritis nodosa, intravenous immunoglobulin, refractory, vasculitis

## Abstract

**Background:**

Cutaneous polyarteritis nodosa (cPAN) is a rare necrotizing vasculitis affecting medium‐sized skin vessels and may be refractory to standard immunosuppressive therapies.

**Case Presentation:**

We report a 23‐year‐old male with HLA‐B27–positive enthesitis‐associated arthritis (ERA), which was reclassified during the disease course and is currently in clinical remission under interleukin‐17 inhibition, and reading epilepsy, presenting with histologically confirmed cPAN. Despite multiple treatments, including methotrexate, corticosteroid pulses, tumor necrosis factor‐α (TNF‐α) inhibitors, and tocilizumab, the patient developed persistent painful livedo reticularis‐like lesions with palpable subcutaneous nodules and systemic symptoms. Intravenous immunoglobulin (IVIG) was initiated at 1.5 g/kg per cycle (administered over three consecutive days; total dose ∼100 g per cycle) and repeated every eight weeks, followed by a clinically meaningful response after the first cycle and complete resolution of symptoms within one month.

**Conclusion:**

The addition of IVIG may represent an effective escalation option for refractory cPAN. Further studies are needed to clarify its role in this rare vasculitis.

## 1. Introduction

Polyarteritis nodosa (PAN) is a necrotizing vasculitis of medium‐sized arteries, with an estimated annual incidence of 0.7 per million children and adolescents [1]. cPAN, termed cutaneous arteritis (CA) in the revised Chapel Hill Consensus Conference nomenclature, is classified as a single‐organ vasculitis and may involve medium‐sized vessels of the skin and adjacent subcutis and muscle [2]. Clinical features can include fever, weight loss, arthralgia, painful livedo reticularis, and subcutaneous nodules, and diagnosis requires biopsy of nodular skin lesions. Antineutrophil cytoplasmic antibodies (ANCA) are typically absent in cPAN [1].

The pathophysiology of cPAN is complex and incompletely understood, involving genetic factors and autoimmune mechanisms; deficiency of adenosine deaminase 2 (ADA2) is an important differential diagnosis [1]. Current treatment recommendations are largely based on consensus and older evidence [1]. Standard approaches include glucocorticoids and, in selected cases, cyclophosphamide for acute management, as well as methotrexate, mycophenolate mofetil, or azathioprine for maintenance [1]. Biologics such as TNF‐α inhibitors and interleukin‐6 blockade, as well as emerging Janus kinase inhibitors, have been reported as options in refractory disease [1, 3]. Sporadic reports also describe rituximab or intravenous immunoglobulin (IVIG) in difficult‐to‐treat cases [4, 5].

## 2. Case Report

### 2.1. Patient Information

We present a 23‐year‐old male student with chronic inflammatory arthritis, recently reclassified as HLA‐B27–positive enthesitis‐associated arthritis, currently in remission under interleukin‐17 inhibition, and reading epilepsy well controlled with lamotrigine.

### 2.2. Main Concerns and Key Clinical Findings

The patient presented with a long‐standing history (since the age of 15 years) of persistent severe pain associated with livedo reticularis‐like skin changes and palpable subcutaneous nodules, predominantly affecting the lower extremities (Figure 1). Symptoms were accompanied by arthralgia, myalgia, marked fatigue, and sleep disturbance, leading to substantial impairment of quality of life.

**FIGURE 1:**
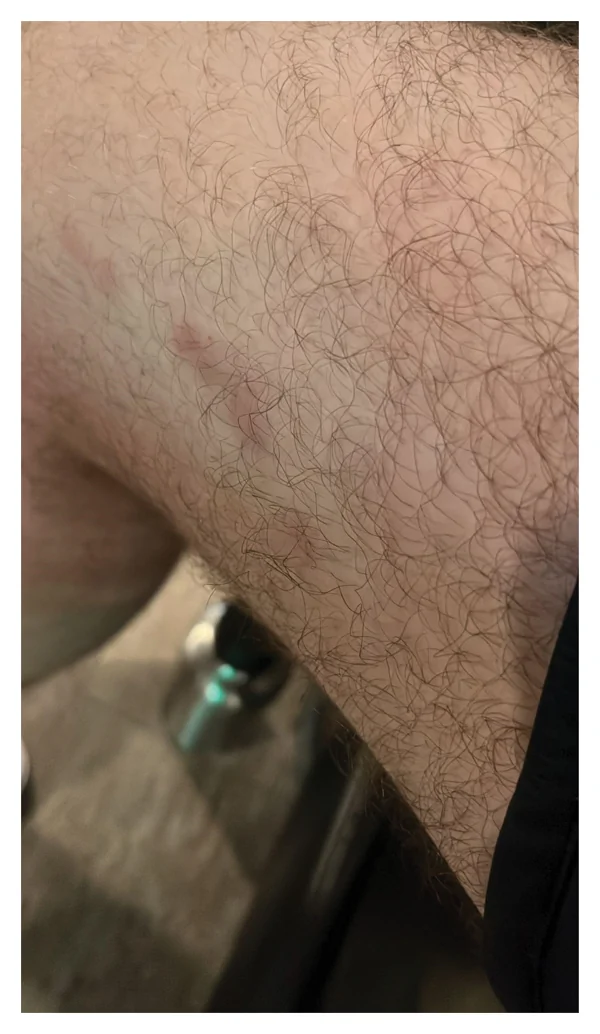
Palpable, painful nodules, and livedo reticularis‐like skin lesions on the right inner thigh (lower extremities) approximately 5 months before initiation of IVIG therapy. Photograph provided by the patient with informed consent.

At the age of 16 years, reading epilepsy was diagnosed. Neurological evaluation, including cerebral magnetic resonance imaging (MRI), revealed no evidence of structural abnormalities or central nervous system involvement.

### 2.3. Diagnostic Assessment

Histopathology from a biopsy of the right calf demonstrated medium‐sized vessel involvement at the dermal–subcutaneous junction and within the subcutaneous fat, with marked luminal narrowing to complete occlusion, fibrin deposition, erythrocyte extravasation, and neutrophilic debris within the vessel wall (Figures 2, 3, and 4). Surrounding tissue showed inflammatory infiltrates and hemorrhage in fibrotically thickened septa. No evidence of malignancy was identified. The diagnosis fulfilled the EULAR/PRINTO/PRES classification criteria for childhood cPAN, with histopathological confirmation of necrotizing medium‐vessel vasculitis (mandatory criterion) together with characteristic cutaneous manifestations (livedo reticularis‐like skin changes and painful subcutaneous nodules) and myalgia [6].

**FIGURE 2:**
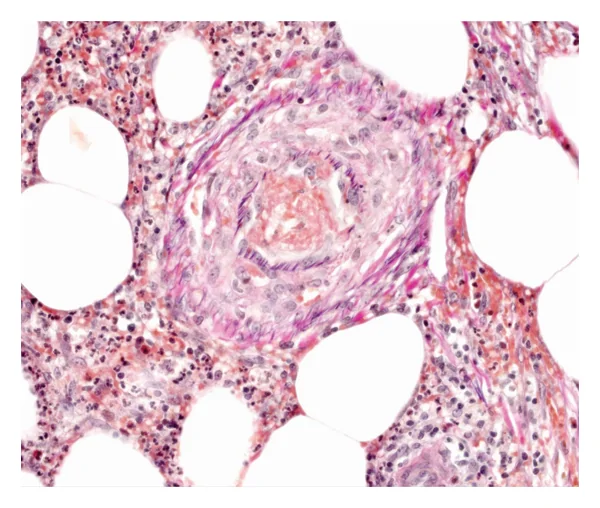
Histopathology of the dorsal aspect of the right lower leg: demonstrating fragmentation of the internal elastic lamina of a small artery. (elastic van Gieson staining, magnification × 100) (courtesy of PD Dr. med. C. Rose, dermatopathology Lübeck).

**FIGURE 3:**
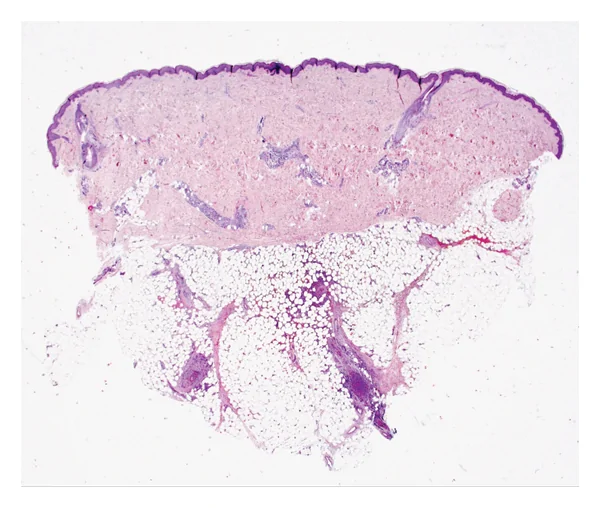
Overview image of a deep skin biopsy showing dilated blood vessels in the adipose tissue (haematoxylin–eosin staining, magnification × 40) (courtesy of PD Dr. med. C. Rose, dermatopathology Lübeck).

**FIGURE 4:**
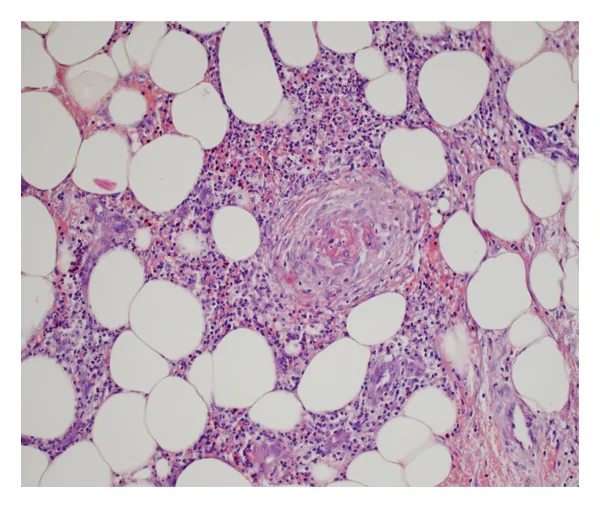
Close‐up of the vasculitis from the centre of the biopsy specimen. Fibrin in the lumen of the small artery and dense vascular and perivascular inflammatory infiltrate containing numerous neutrophils (haematoxylin and eosin staining, magnification × 100) (courtesy of PD Dr. med. C. Rose, dermatopathology Lübeck).

During follow‐up, the patient developed reading epilepsy, prompting neurological evaluation including cranial MRI. The MRI revealed no structural abnormalities or findings suggestive of central nervous system vasculitis. Apart from this neurological manifestation, no clinical signs of systemic vasculitic involvement, including renal, gastrointestinal, or peripheral vascular manifestations, were identified. Therefore, in the absence of evidence suggesting systemic arterial involvement, further vascular imaging was not pursued.

During disease flares, laboratory investigations showed a fluctuating inflammatory profile, with CRP levels up to 82.7 mg/L (normal < 5 mg/L), ESR up to 66 mm/h, ferritin up to 427 µg/L (normal 6–70 µg/L), and LDH up to 1098 U/L (normal < 235 U/L). Serological testing, including hepatitis B and C serology, as well as ANA and ANCA, was negative.

Whole‐genome sequencing did not identify pathogenic variants explaining the clinical phenotype, including variants associated with monogenic vasculitic or autoinflammatory diseases such as ADA2 deficiency. A heterozygous MVK variant (MVK V377I) was detected; however, clinical and genetic criteria for mevalonate kinase deficiency (hyper‐IgD syndrome) were not fulfilled [7].

### 2.4. Diagnostic Challenges

Diagnostic work‐up was challenging due to the initially established diagnosis of systemic juvenile idiopathic arthritis (sJIA), which complicated the interpretation of cutaneous manifestations and contributed to diagnostic uncertainty regarding their attribution to a vasculitic process. The initially subclinical and relapsing disease course, with intermittent improvement of symptoms and inflammatory markers, further delayed definitive diagnosis.

### 2.5. Differential Diagnoses

Differential diagnoses included systemic PAN, deficiency of ADA2 (DADA2), livedoid vasculopathy and other thrombotic/vasculopathic disorders (including antiphospholipid syndrome), infection‐related vasculitis, and other medium‐vessel vasculitides. The persistent skin‐limited phenotype, repeated compatible histopathology, and negative genetic testing for ADA2 deficiency supported idiopathic cPAN.

### 2.6. Therapeutic Interventions Prior to IVIG

Initially, the patient showed a good clinical response to oral prednisolone (15 mg). However, over the subsequent disease course, clinical improvement could only be achieved with repeated high‐dose intravenous methylprednisolone pulse therapies (3 consecutive days, cumulative dose of 3000 mg per cycle). Given the potential adverse effects associated with prolonged high‐dose glucocorticoid exposure, long‐term corticosteroid therapy was not considered a suitable maintenance strategy.

Over the years, the patient received multiple immunomodulatory and immunosuppressive therapies, including methotrexate, repeated intravenous methylprednisolone pulses during disease flares, dapsone, colchicine, tocilizumab, mycophenolate mofetil, adalimumab, etanercept, and azathioprine, without achieving sustained clinical remission (Figure 5).

**FIGURE 5:**
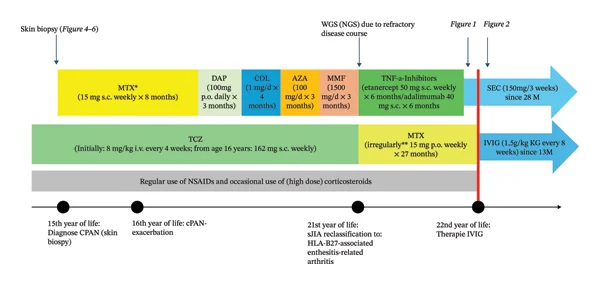
Timeline summarizing disease course, diagnostic evaluation, prior therapies, and response to intravenous immunoglobulin (IVIG). ∗initially, methotrexate (MTX) showed good clinical efficacy. However, treatment had to be discontinued because of adverse effects (nausea and fatigue). Given the favorable therapeutic response, several subsequent attempts to reintroduce MTX were made by the paediatric rheumatology team, but each was discontinued because of recurrent adverse effects. ∗∗following initiation of TNF‐α inhibitor therapy, the adult rheumatology team made another attempt to reintroduce MTX. The patient later reported taking the medication only intermittently because of the same adverse effects described above. After this additional attempt was also unsuccessful, no further MTX rechallenges were undertaken. AZA: azathioprine, COL: colchicine, DAP: Dapson, IVIG: intravenous immunoglobulin, MMF: mycophenolate mofetil, MTX: methotrexate, NGS: next‐generation sequencing, NSAID: nonsteroidal anti‐inflammatory drugs, SEC: secukinumab, TCZ: tocilizumab, TNF‐α: tumor necrosis factor alpha, WGS: whole‐genome sequencing.

### 2.7. Therapeutic Intervention and Follow‐Up/Outcomes

As a last resort, IVIG therapy was initiated at a dose of 1.5 g/kg body weight per cycle. IVIG was administered over three consecutive days (total dose ∼100 g per cycle) and repeated every eight weeks. A clinically meaningful response was already evident after the first cycle, and the patient achieved complete resolution of painful skin lesions and associated systemic symptoms within one month (Figure 6). Treatment was well tolerated, and no adverse events were reported. Concomitant interleukin‐17 inhibition for the patient’s inflammatory arthritis was continued unchanged during IVIG therapy. At last follow‐up, 19 months after IVIG initiation, the patient remained clinically stable without recurrence of painful cutaneous lesions. The longitudinal disease course and treatments are summarized in Figure 5.

**FIGURE 6:**
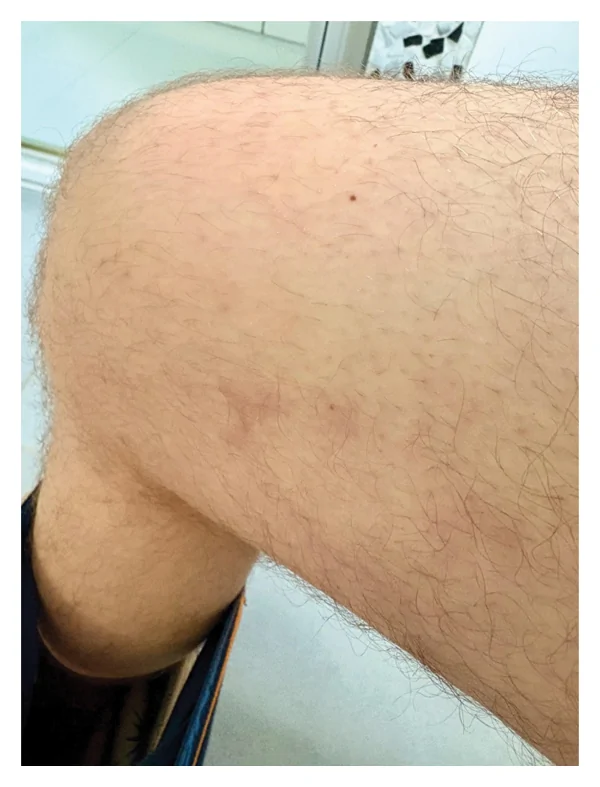
Skin of the right inner thigh after the third cycle of IVIG therapy, showing marked resolution of livedo reticularis‐like changes. No palpable, painful nodules are visible. Photograph provided by the patient with informed consent.

## 3. Discussion

The pathogenesis of cPAN is thought to be driven predominantly by dysregulated innate immune activation. Although ANCA is typically absent in cPAN, other circulating autoantibodies have been reported in subsets of patients. Antiphosphatidylserine/prothrombin complex (anti‐PS/PT) antibodies were frequently detected in a small cohort of patients with cPAN, with IgM anti‐PS/PT levels correlating with CRP levels in LAC‐positive patients, while more recent proteome‐wide screening identified antitranscobalamin receptor (anti‐TCblR) antibodies in a subset of patients with CA, with potential diagnostic and pathophysiological significance [8, 9]. Localized immune complex deposition has also been demonstrated within affected vessels. Histologically, vascular lesions are dominated by macrophages together with innate‐like bystander‐activated CD8^+^ T cells, supporting the concept that innate immune effector mechanisms play a central role in disease pathogenesis [10].

These immunopathological features provide a biologically plausible rationale for IVIG therapy. IVIG interacts with Fcγ receptors expressed on macrophages, thereby modulating downstream inflammatory signaling. Given that macrophages constitute the predominant inflammatory cell population in cPAN lesions, modulation of macrophage activation may represent one potential mechanism underlying the rapid clinical response observed in our patient. In addition, IVIG exerts broader immunomodulatory effects, including regulation of cytokine networks and immune complex‐mediated inflammation [11].

Although IVIG is not considered standard therapy for cPAN, its use in refractory disease is biologically plausible. Previous reports have described beneficial effects of IVIG in selected patients with refractory PAN/cPAN. In a cohort of 33 patients with CA, Matsuda et al. identified male sex, skin ulceration, and elevated CRP levels as factors associated with treatment resistance and the need for more intensive therapy. Notably, our patient was male and exhibited markedly elevated CRP levels during disease flares, findings that are consistent with features associated with treatment resistance in this cohort [12].

In our patient, the rapid clinical improvement following IVIG initiation supports its consideration as an escalation option after failure of conventional immunosuppressive and biologic therapies. However, secukinumab was continued concomitantly and unchanged throughout IVIG treatment. Therefore, a delayed therapeutic effect of secukinumab or a combined effect of both therapies cannot be excluded, and the observed clinical improvement cannot be attributed solely to IVIG. Nevertheless, the close temporal association between IVIG initiation and clinical improvement suggests a potential therapeutic contribution of IVIG. Moreover, evidence regarding IVIG in cPAN remains limited and is currently based mainly on case reports and small case series. Further studies are required to define optimal timing, patient selection, and long‐term efficacy of IVIG in refractory cPAN.

## 4. Conclusion

This case illustrates that cPAN may remain strictly skin‐limited yet cause profound pain and functional impairment despite multiple immunosuppressive and biologic therapies. The addition of IVIG may represent an escalation option in refractory cPAN and was associated with rapid clinical improvement and good tolerability in this case. Careful diagnostic work‐up, including exclusion of monogenic vasculitis mimics such as ADA2 deficiency and assessment for systemic involvement, is essential. Prospective documentation of patient‐reported outcomes and longer follow‐up will help define the role of IVIG in refractory cPAN.

## Funding

This research received no external funding.

## Disclosure

Fabio Kohlmann is a medical student with a research interest in rheumatology and rare inflammatory diseases.

## Ethics Statement

Ethical approval was not required for this case report in accordance with local and institutional guidelines.

## Consent

Written informed consent for publication of this case report and the accompanying images was obtained from the patient.

## Conflicts of Interest

The authors declare no conflicts of interest.

## Data Availability

Data sharing is not applicable to this article as no datasets were generated or analyzed during the current study.

## References

[1] de Graeff N. , Groot N. , Brogan P. et al., European Consensus-based Recommendations for the Diagnosis and Treatment of Rare Paediatric Vasculitides–The SHARE Initiative, Rheumatology. (2019) 58, no. 4, 656–671, 10.1093/rheumatology/key322. 30535249

[2] Jennette J. C. , Falk R. J. , Bacon P. A. et al., Revised International Chapel Hill Consensus Conference Nomenclature of Vasculitides, Arthritis & Rheumatism. (2013) 65, no. 1, 1–11, 10.1002/art.37715. 23045170

[3] Conticini E. , Sota J. , Falsetti P. et al., Biologic Drugs in the Treatment of Polyarteritis Nodosa and Deficit of Adenosine Deaminase 2: A Narrative Review, Autoimmunity Reviews. (2021) 20, no. 4, 10.1016/j.autrev.2021.102784. 33609794

[4] Boukhris I. , Hamdi M. S. , Hariz A. et al., Efficacy of Rituximab in Refractory Polyarteritis Nodosa: A Case Report, Pan Afr Med J. (2023) 45, 10.11604/pamj.2023.45.92.36496. PMC10491718 37692987

[5] Turska M. and Parada-Turska J. , [Cutaneous Polyarteritis Nodosa], Wiadomosci Lekarskie. (2018) 71, no. 1 Pt 1, 73–77. 29558355

[6] Ozen S. , Pistorio A. , Iusan S. M. et al., EULAR/PRINTO/PRES Criteria for Henoch-Schönlein Purpura, Childhood Polyarteritis Nodosa, Childhood Wegener Granulomatosis and Childhood Takayasu Arteritis: Ankara 2008. Part II: Final Classification Criteria, Annals of the Rheumatic Diseases. (2010) 69, no. 5, 798–806, 10.1136/ard.2009.116657. 20413568

[7] Gattorno M. , Hofer M. , Federici S. et al., Classification Criteria for Autoinflammatory Recurrent Fevers, Annals of the Rheumatic Diseases. (2019) 78, no. 8, 1025–1032, 10.1136/annrheumdis-2019-215048. 31018962

[8] Kawakami T. , Yamazaki M. , Mizoguchi M. , and Soma Y. , High Titer of Anti-Phosphatidylserine-Prothrombin Complex Antibodies in Patients With Cutaneous Polyarteritis Nodosa, Arthritis & Rheumatism. (2007) 57, no. 8, 1507–1513, 10.1002/art.23081. 18050170

[9] Matsuda K. M. , Kotani H. , Yamaguchi K. et al., Significance of Anti-Transcobalamin Receptor Antibodies in Cutaneous Arteritis Revealed by Proteome-Wide Autoantibody Screening, Journal of Autoimmunity. (2023) 135, 10.1016/j.jaut.2023.102995. 36724643

[10] Benseler S. , Wagner N. , Dannecker G. , and Kallinich T. , Polyarteriitis Nodosa, Eosinophile Granulomatose Mit Polyangiitis (Churg-Strauss-Syndrom) Und Andere Seltene Vaskulitiden Bei Kindern, Pädiatrische Rheumatologie. Springer Reference Medizin, 2022, Springer, Berlin, Heidelberg, 10.1007/978-3-662-60411-3_49-1.

[11] Kazatchkine M. D. , Bellon B. , and Kaveri S. V. , Mechanisms of Action of Intravenous Immunoglobulin (IVIG), Multiple Sclerosis. (2000) 6, no. Suppl 2, S24–S26, 10.1177/135245850000602s07. 11188775

[12] Matsuda K. M. , Yoshizaki A. , Kotani H. et al., Development of a Prediction Model of Treatment Response in Patients With Cutaneous Arteritis: Insights From a Cohort of 33 Patients, The Journal of Dermatology. (2021) 48, no. 7, 1021–1026, 10.1111/1346-8138.15868. 33768589

